# Cangrelor ameliorates CLP-induced pulmonary injury in sepsis by inhibiting GPR17

**DOI:** 10.1186/s40001-021-00536-4

**Published:** 2021-07-06

**Authors:** Qiancheng Luo, Rui Liu, Kaili Qu, Guorong Liu, Min Hang, Guo Chen, Lei Xu, Qinqin Jin, Dongfeng Guo, Qi Kang

**Affiliations:** 1 Department of Critical Care Medicine, Shanghai Gongli Hospital, The Second Military Medical University, Shanghai, 200135 People’s Republic of China; 2 Department of Endocrinology, Shanghai Gongli Hospital, The Second Military Medical University, Shanghai, 200135 People’s Republic of China; 3 Postgraduate Training Base in Shanghai Gongli Hospital, Ningxia Medical University, Shanghai, 200135 People’s Republic of China

**Keywords:** Sepsis, Inflammation, Cangrelor, Platelet, GPR17

## Abstract

**Background:**

Sepsis is a common complication of severe wound injury and infection, with a very high mortality rate. The P2Y12 receptor inhibitor, cangrelor, is an antagonist anti-platelet drug.

**Methods:**

In our study, we investigated the protective mechanisms of cangrelor in CLP-induced pulmonary injury in sepsis, using C57BL/6 mouse models.

**Results:**

TdT-mediated dUTP Nick-End Labeling (TUNEL) and Masson staining showed that apoptosis and fibrosis in lungs were alleviated by cangrelor treatment. Cangrelor significantly promoted surface expression of CD40L on platelets and inhibited CLP-induced neutrophils in Bronchoalveolar lavage fluid (BALF) (*p* < 0.001). We also found that cangrelor decreased the inflammatory response in the CLP mouse model and inhibited the expression of inflammatory cytokines, IL-1β (*p*  < 0.01), IL-6 (*p* < 0.05), and TNF-α (*p* < 0.001). Western blotting and RT-PCR showed that cangrelor inhibited the increased levels of G-protein-coupled receptor 17 (GPR17) induced by CLP (*p* < 0.001).

**Conclusion:**

Our study indicated that cangrelor repressed the levels of GPR17, followed by a decrease in the inflammatory response and a rise of neutrophils in BALF, potentially reversing CLP-mediated pulmonary injury during sepsis.

## Background

Sepsis is a serious disease and will lead a high mortality rate of approximately 22% in all over the world [1]. It has several features like out of controlled inflammatory response, coagulation disorder, and immune dysfunctions [2]. The cause of sepsis is complex, and its main cause is a response caused by the host’s immune response to the pathogen [2, 3]. Overall, the physiological and pathological mechanisms of sepsis are well researched. A lot of mediators such as cytokines, chemokines, and free radicals participate in the development of sepsis [4]. The lung is one of the initial target organ of the systemic inflammatory response caused by sepsis, leading to alveolar or capillary epithelial cell injury, diffuse pulmonary interstitial edema, and inflammatory cell exudation (i.e., acute lung injury/respiratory distress syndrome, progressive respiratory distress, and hypoxemia) and then acute respiratory failure and non-cardiogenic pulmonary edema [5–8]. Despite numerous treatment strategies, drugs that effectively improve the treatment of sepsis remain to be developed. Antibiotics are the key method for clinical treatment, but the death rate of sepsis still reaches an astonishing number of 30–40% [9].

In the past few years, the treatment of sepsis-related coagulopathy has been widely investigated. Recent studies have found that there is a coagulation–inflammation cross-reaction system with platelet–leukocyte aggregates as the core pathogenesis of sepsis [10]. With inhibition of platelet activation by anti-platelet drugs being used to treat sepsis/acute lung injury, the high risk of patients could be reduced organ damage and alleviated conditions [11, 12]. Former studies have found that P2Y12 receptor antagonists such as clopidogrel and ticagrelor treat sepsis and lung damage through anti-platelet aggregation effects [13–15]. The P2Y12 receptor belongs to a protein-coupled receptor and is one of two receptors on platelets. The P2Y12 receptor is also the initiation channel for platelet activation, and the receptor strongly amplifies signal activation. This amplification is displayed not only in the magnification of the platelet aggregation signal, as well as in subsequent multi-step signaling, including the release of platelet particles and the enhancement of platelet-promoting activity [16, 17]. P2Y12 receptor antagonists prevent the formation of neutrophil–platelet aggregates by inhibiting the recruitment of neutrophils, significantly reducing thrombocytopenia, and also reducing lung damage caused by sepsis [18–20]. However, the therapeutic mechanism of cangrelor on sepsis has not been reported. Our research aims were to characterize the potential effect of cangrelor on sepsis and to identify its mechanism of action.

## Materials and methods

### Drugs and reagents

Cangrelor (98% pure) was obtained from Vcare PharmaTech Co., Ltd (Shanghai, China). Myeloperoxidase (MPO) kit was obtained from Nanjing Jiancheng (Jiangsu, China). BCA Protein Assay was obtained from Thermo Fisher (MA, USA). Anti-CD40L (#15094, 1:1000) and β-actin (#4970, 1:1000) were obtained from Cell Signalling Technology (MA, USA). Anti-GPR17 (ab75553, 1:500) was obtained from Abcam (Shanghai, China). Enzyme-linked immunosorbent assay (ELISA) kits were obtained from RapidBio (CA, USA). TRIzol reagent was obtained from Life Technology (CA, USA). PrimeScript^®^ RT Master Mix and SYBR^®^ Premix Ex Taq™ were purchased from Takara (Dalian, China). All other reagents were obtained from Sigma-Aldrich (Shanghai, China).

### Animal treatment

C57BL/6 mice (20 ± 2 g) were purchased from Shanghai Laboratory Animal Center (Shanghai, China) and fed according to guidelines approved by the Experimental Animal Ethical Committee of Pudong New Area Gongli Hospital. They were raised in a constant temperature and humidity room (22 ± 1 °C, 65 ± 5% humidity) with standard diet and water in Pudong New Area Gongli Hospital. For the survival study, 62 mice were randomly separated into 4 groups: (1) sham (*n* = 8), (2) cecal ligation and double puncture (CLP) (*n* = 18), (3) CLP + cangrelor (5 mg/kg) (*n* = 18), and (4) CLP + cangrelor (20 mg/kg) (*n* = 18). Another 32 mice were randomly separated into 4 groups: (1) sham (*n* = 8), (2) cecal ligation and double puncture (CLP) (*n* = 8), (3) CLP + cangrelor (5 mg/kg) (*n* = 8), and (4) CLP + cangrelor (20 mg/kg) (*n* = 8). The CLP was performed on isoflurane-anesthetized mice as previously described [21, 22]. Sham control animals underwent a laparotomy without ligation or double puncture. Mice were orally administrated with cangrelor (5 or 20 mg/kg) 2 h before surgery. After surgery but prior to emergence, mice were fluid-resuscitated (1 mL/mouse of sterile saline, subcutaneously). For the mechanism study, 32 mice were randomly separated into four groups: (1) sham (*n* = 8), (2) cecal ligation and double puncture (CLP) (*n* = 8), (3) CLP + cangrelor (5 mg/kg) (*n* = 8), and (4) CLP + cangrelor (20 mg/kg) (*n* = 8). Mice were killed 24 h after induction of CLP, plasma and lung were saved.

### Masson staining of the lung

Paraffin sections of lung tissues were analyzed after several methods containing dewaxing rehydration, Ponceau S Fuchs in acid solution staining for 300 s, by washing in 1% phosphomolybdic acid differentiation solution for 5 min, aniline blue solution counterstaining for 300 s, with 1% glacial acetic acid for 1 min, alcohol gradient dehydration, transparency production using dimethylbenzene and xylene, and mounting.

### Flow cytometry

For detection of surface CD40L expression on platelets, plasma was placed for 10 min at 36.5 °C that blocked FcγIII/II receptors to decrease nonspecific labeling, next incubated with fluorescein isothiocyanate-conjugated anti-CD41. Immobilization of cells with 1% formaldehyde; erythrocytes were lysed with fluorescence-activated cell sorting lysing solution (BD Biosciences, Shanghai, China), and neutrophils and platelets were then recovered by centrifugation at 300×*g* for 10 min. Platelet activation was analyzed using PE‐conjugated JON/A antibody (Emfret Analytics, Würzburg, Germany), which bound to the high affinity conformation of mouse αffini.

### RNA isolation and quantitative real-time PCR (qRT-PCR)

mRNA was obtained using TRIzol, respectively. cDNA was synthesized using M-MLV reverse transcriptase and Sangon reverse transcription primers. Relative transcript abundance was determined by the 2^−ΔΔCt^ method and mRNA expression was normalized against β-actin. All amplification analyses were completed on QuantStudio™ 6 Flex Real-Time PCR System.

### Protein extraction and western blot analysis

Lung tissues were lysed by RIPA buffer and protein concentrations were assessed by the BCA protein assay. About 20 μg of protein from each example was divided by a 10% SDS–polyacrylamide gel and transferred to polyvinylidene fluoride membranes. Membranes were blocked with 10% skim milk in Tris-Buffered Saline with Tween-20 (TBST) and incubated with primary antibodies overnight at 4 °C. Membranes were incubated with the corresponding secondary antibody for 1 h at room temperature and washed in TBST. Protein signals were detected using the Super ECL Plus Detection Reagent.

### Enzyme-linked immunosorbent assay (ELISA)

Concentrations of the inflammatory cytokines in mice serum were measured with an ELISA kit following to manufacture protocols.

### Statistical analysis

The effects of Cangrelor on the survival of mice were analyzed using the Kaplan–Meier curve and log-rank test. All other results were analyzed using one-way ANOVA analysis by SPSS statistical software for Windows, version 22.0 (SPSS, Chicago, IL, USA). The measurement data are expressed as the mean ± SD. A value of *p* < 0.05 was deemed statistically significant.

## Results

### Cangrelor alleviates CLP-induced pulmonary injury

**Fig. 1:**
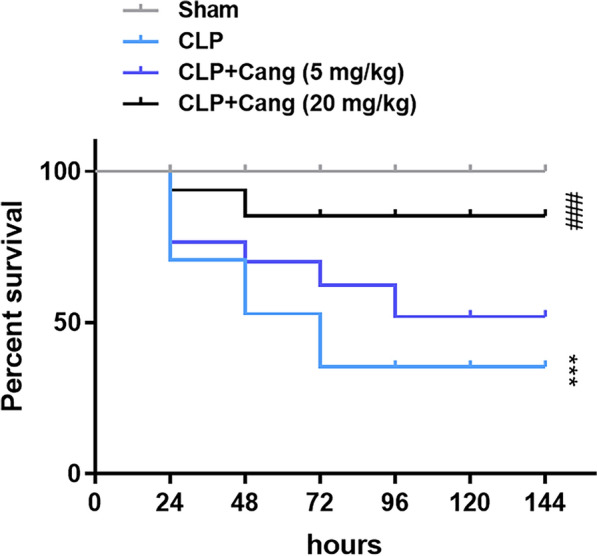
Cangrelor improved the survival rate of sepsis mice. *n* = 8 for control group and *n* = 18 for all other groups

**Fig. 2:**
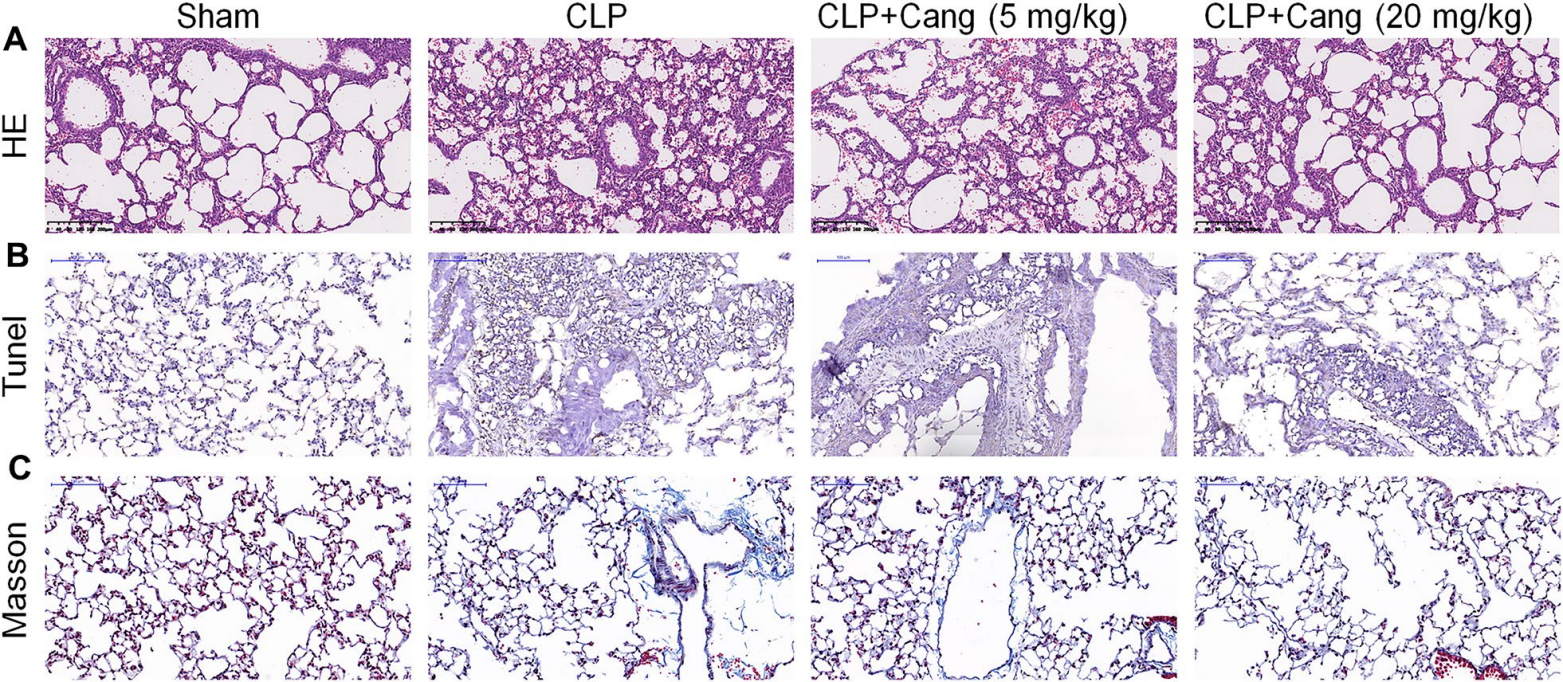
Cangrelor alleviates CLP-induced pulmonary injury. **A** Representative images of HE of mouse lung sections. **B** Representative images of TUNEL staining of mouse lung sections. **C** Representative images of Masson staining of mouse lung sections

First, as shown in Fig. 1, the survival rate was 72.22% on the first 24 h, decreased to 55.56% on the next 24 h, and decreased a stable level of 38.89% on 72 h after CLP (*p* < 0.001). Treating with 5 mg/kg cangrelor did not enhance the survival rate compared with CLP group, while treating with 20 mg/kg cangrelor significantly improved the survival rate compared with CLP group (*p* < 0.001). Next, HE staining showed noticeable histopathologic changes including congestion, inflammatory cell infiltration, necrosis and degeneration while treating with 20 mg/kg cangrelor significantly ameliorate the histopathologic changes induced by CLP (Fig. 2A). In addition, we assessed the levels of apoptosis in mice lung tissues, using a TUNEL assay. The results showed that in the CLP group, lung tissues had a higher level of apoptotic cells, but cangrelor (20 mg/kg) reversed this result (Fig. 2B). Next, we assessed the anti-inflammatory effect of cangrelor. Masson staining showed that CLP mice had fibrotic changes, while there was significantly less damage in the CLP + cangrelor (20 mg/kg) group, as showed by blue staining (Fig. 2C).

### Cangrelor regulates platelet aggregation and neutrophil recruitment.

**Fig. 3:**
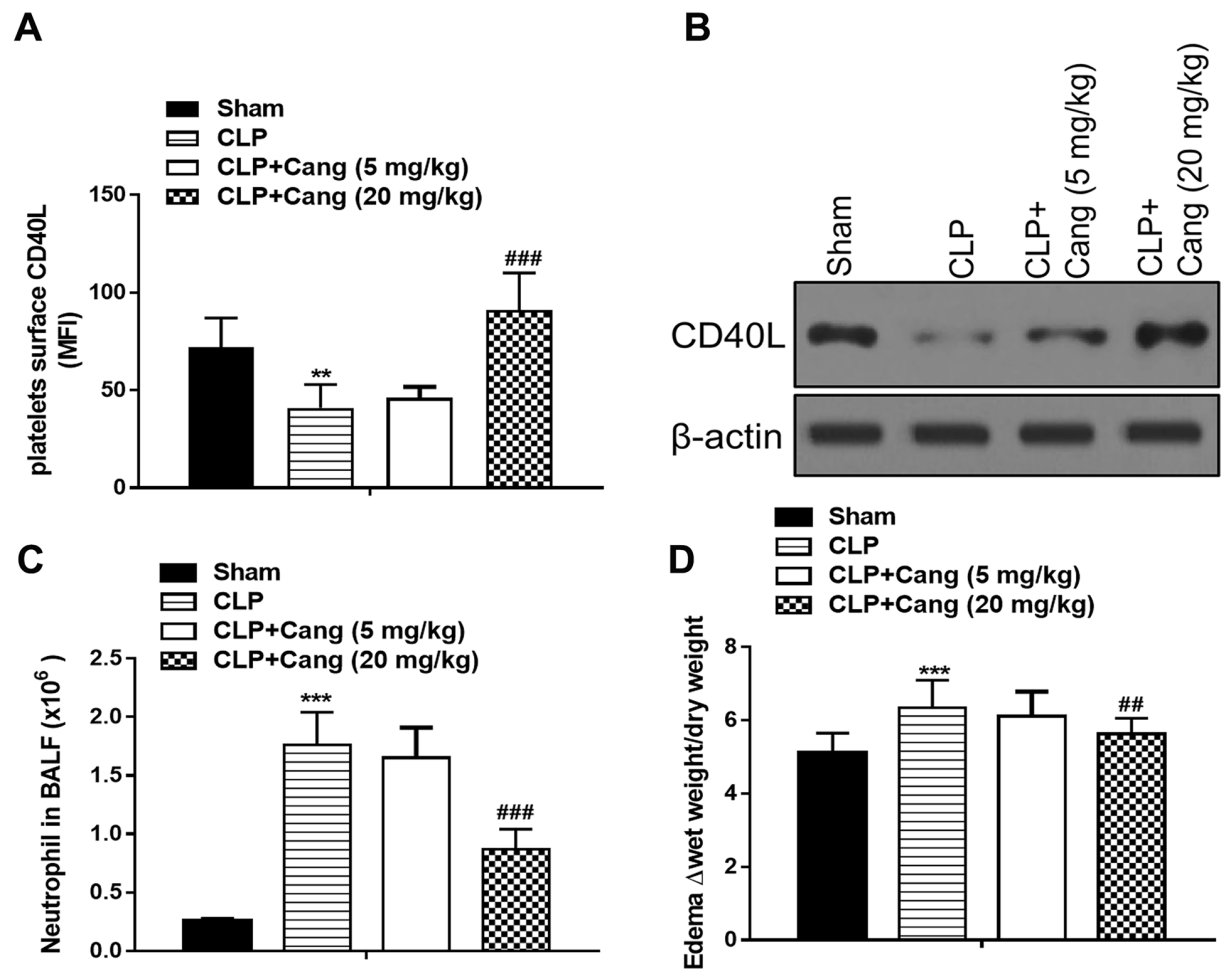
Cangrelor regulates platelet aggregation and neutrophil recruitment. **A** Platelet surface expression CD40L. **B** Western blots of platelet lysates CD40L. **C** The number of neutrophils in BALF. **D** The levels of edema formation in the lung. Results are presented as means ± SD (*n* = 8). ^**^p < 0.01, ^***^ p < 0.001 compared to the sham control; ^##^*P* < 0.01, ^###^*P* < 0.001 compared to CLP

The platelets surface level of CD40L was measured (Fig. 3A). The mean fluorescence intensity (MFI) was 72 in the sham group and 41 in the CLP group (*p* < 0.01). After the administration of cangrelor, the MFI was 46 when treated with 5 mg/kg cangrelor and 91 when treated with 20 mg/kg cangrelor which was significantly increased compared with CLP group (*p* < 0.001). Western blotting also presented that the level of platelet CD40L was meaningfully decreased in the CLP group in contrast to the sham group, while cangrelor (20 mg/kg) increased this reduction (Fig. 3B). A cell assay of the broncho-alveolar lavage fluid (BALF) indicated the neutrophils in the broncho-alveolar was 0.26 × 10^6^ in the sham mice while it was 1.76 × 10^6^ when induced by CLP. After cangrelor treatment, the number of neutrophils decreased to 0.87 × 10^6^ (*p* < 0.001) (Fig. 3C). In addition, the pulmonary wet/dry ratio in the CLP group increased, which indicated the formation of pulmonary edema (*p* < 0.001). However, treatment with 20 mg/kg cangrelor significantly reversed this edema compared with CLP group (*p* < 0.01) (Fig. 3D).

### Cangrelor ameliorates the inflammatory response through GPR17

**Fig. 4:**
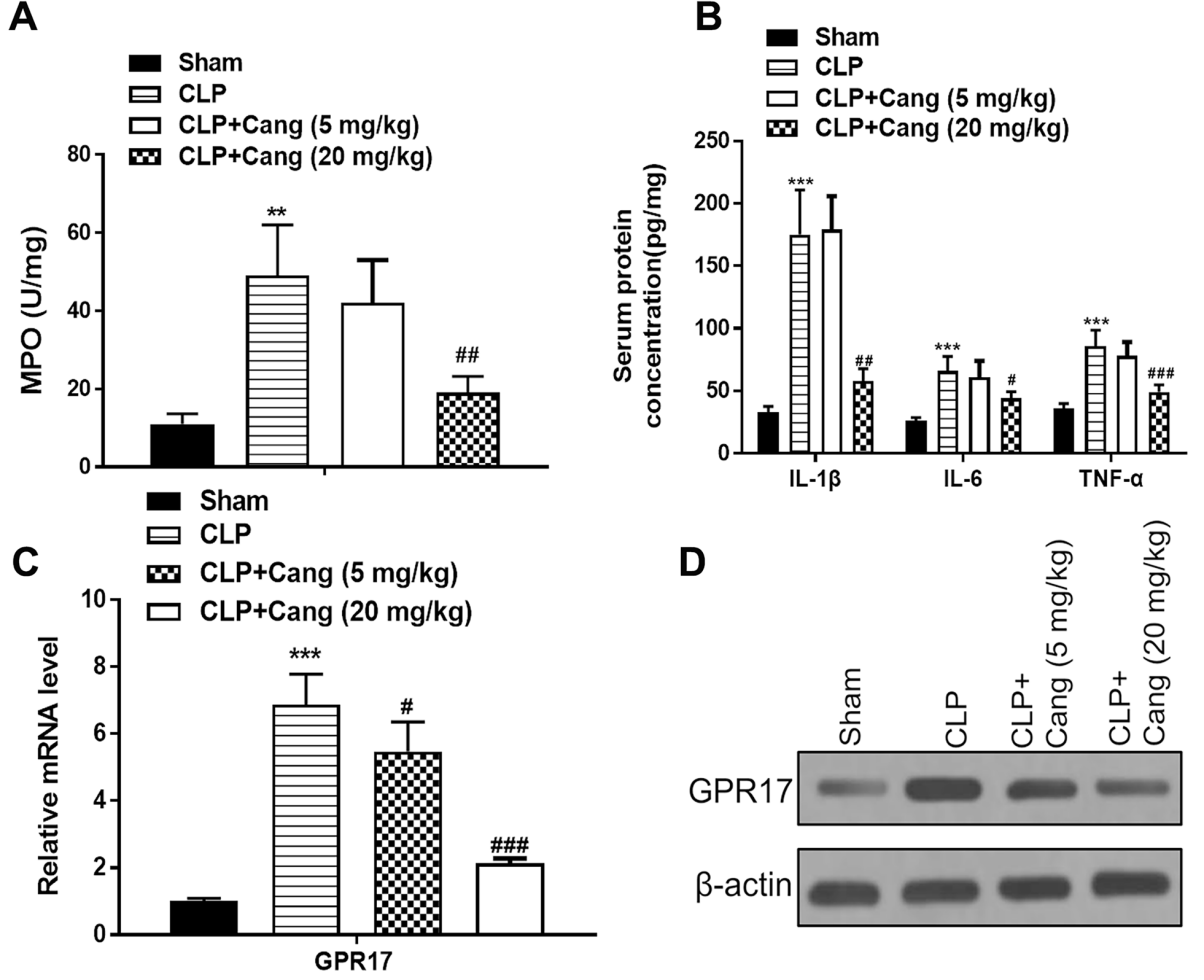
Cangrelor ameliorates the inflammatory response through GPR17. **A** MPO concentration in lung tissue. **B** Serum concentrations of TNF-α, IL-1β, and IL-6 (**C**) mRNA level of GPR17. **D** Western blot of GPR17 in lung tissue. Results are presented as means ± SD (*n* = 8). ^**^p < 0.01, ^***^p < 0.001 compared to the sham control; ^##^*p* < 0.01, ^###^*p* < 0.001 compared to CLP

CLP-induced levels of MPO in mice lung tissues were significantly increased (*p* < 0.01), whereas they decreased significantly in the presence of cangrelor (*p* < 0.01) (Fig. 4A). Using an ELISA, the concentrations of TNF-α, IL-1β, and IL-6 in mice serum were meaningfully raised in the presence of CLP (all *p* < 0.001), however, these levels were reduced by cangrelor (20 mg/kg) (IL-1β: *p* < 0.01, TNF-α: *p* < 0.05, IL-6: *p* < 0.001) (Fig. 4B). Former research has reported that cangrelor is the antagonist of GPR17 [23]. As shown in Fig. 4C, D western blotting and RT-PCR results indicated CLP upregulated GPR17 level in lung tissues(*p* < 0.001), whereas the administration of cangrelor (20 mg/kg) decreased its expression(*p* < 0.001).

## Discussion

In our research, we demonstrated that cangrelor alleviated CLP-induced lung injury in abdominal sepsis by regulating platelet aggregation and the inflammatory response.

Previous studies reported that P2Y12 receptor antagonists such as clopidogrel and ticagrelor alleviate sepsis and lung damage through anti-platelet aggregation [13–15]. In addition, increasing evidence has shown that P2Y12 receptor antagonists decrease the inflammatory response in cardiovascular events, thrombosis, and atherosclerosis [18, 24], which indicates that P2Y12 regulation can affect inflammation through mechanisms that have not yet been fully clarified. Cangrelor is a second-generation P2Y12 antagonist and past studies have found that cangrelor alleviates the inflammatory response in lung fibrosis [25], chronic inflammatory pain [26], and neuropathic pain [27]. In the present study, we found that pre-treatment of cangrelor decreased CLP-induced pulmonary injury by modulating the inflammatory response and significantly increased survival in sepsis mice. However, the diagnosis and treatment of sepsis usually occur after the onset of the disease. Hence, we will further investigate the time responses of cangrelor and evaluated the therapeutic effect of cangrelor at different time after CLP. Besides, lipopolysaccharide (LPS)-stimulated lung epithelial/endothelial cells are commonly used to mimic in vitro sepsis-induced lung injury model [28, 29]. What is more, Amison et al. [30] found that pulmonary neutrophil recruitment induced by intranasal LPS administration was inhibited in mice administered either with P2Y receptor antagonists. Hence, further studies are required to establish the effectiveness of treatment with the P2Y12 receptor antagonists, cangrelor in LPS-induced in vitro model*.*

Previous studies have also reported the release of soluble CD40L from platelets in sepsis-regulated platelet-dependent neutrophil lung accumulation [31, 32]. However, the mechanism for modulating CD40L platelet shedding in sepsis is not clear. In our study, CLP induced a decrease of surface CD40L, which was significantly increased after treatment with cangrelor. In addition, the increase of neutrophils in BALF was attenuated by cangrelor treatment. The increased of lymphocytes, macrophages and eosinophils in BALF also indicates lung damage. The immune regulation of macrophages is also considered to be an important target for sepsis [33]. Further research should be undertaken to investigate the effect of cangrelor on macrophage-specific therapeutic targeting their immunometabolism during sepsis and regulation of macrophage.

Cangrelor has been confirmed to effectively inhibit the expression of GPR17 [23, 34, 35]. There is increasing evidence that the modulation of GPR17 could ameliorate inflammatory responses and injury, which suggested that GPR17 plays an important role in injury reparation [36]. For example, inhibiting GPR17 repressed the activation of microglia and alleviated inflammation induced by ischemic in rats [34, 37]. Inhibition of GPR17 also protects against myocardial fibrosis caused by cardiac ischemia [38], and we further confirmed that the administration of cangrelor reversed the rise of GPR17 induced by CLP, as well as ameliorating the inflammatory response. However, the relationship between P2Y12 receptor and GPR17 is still unknown. Besides, whether cangrelor interacts with GPR17 directly or through other unknown pathways remains unclear. In future research, we will investigate drug binding site of cangrelor on GPR17 via molecular docking or surface plasmon resonance analysis.

## Conclusion

Our study suggested that cangrelor inhibited the expression of GPR17 in lung tissue, thus attuned the inflammatory response and the recruitment of neutrophils in BALF, and ameliorated CLP-mediated pulmonary injury during sepsis. In summary, our findings revealed that cangrelor could ameliorate lung injury and increased survival rate of mice with CLP-induced sepsis. These results suggest that cangrelor could be a potential target treatment for sepsis-induced lung injury.

## Data Availability

The data that support the findings of this study are available from the corresponding author upon reasonable request.

## Abbreviations

BALF: Bronchoalveolar lavage fluid
CLP: Cecal ligation and puncture
GPR17: G-protein-coupled receptor 17
MPO: Myeloperoxidase
MFI: Mean fluorescence intensity
TUNEL: TdT-mediated dUTP nick-end labeling
HE: Staining hematoxylin–eosin staining
BCA: Butyleyanoacrylate
RIPA: Radio immunoprecipitation assay
qRT-PCR: Quantitative reverse transcription polymerase chain reaction
TBST: Tris-buffered saline with tween-20
ELISA: Enzyme-linked immunosorbent assay
LPS: Lipopolysaccharide
